# The power of personas: Exploring an innovative model for understanding stakeholder perspectives in an oncology learning health network

**DOI:** 10.1002/lrh2.10422

**Published:** 2024-05-27

**Authors:** Dylan J. Cooper, Jessie Karten, Sarah E. Hoffe, Daniel A. King, Matthew Weiss, Danielle K. DePeralta, Andrew L. Coveler, Sunil R. Hingorani, Tracey Shefter, Cheryl Meguid, Hannah Roberts, Theodore S. Hong, Amol Narang, Amy Hacker‐Prietz, George A. Fisher, Jay Sandler, Laurie Singer, Bobby Korah, William Hoos, Carrie T. Stricker, Joseph M. Herman

**Affiliations:** ^1^ Northwell New Hyde Park New York USA; ^2^ Moffitt Cancer Center Tampa Florida USA; ^3^ Fred Hutchinson Cancer Center Seattle WA USA; ^4^ University of Colorado School of Medicine Aurora Colorado USA; ^5^ Massachusetts General Hospital Boston MA USA; ^6^ Johns Hopkins University School of Medicine Baltimore Maryland USA; ^7^ Stanford University School of Medicine Stanford California USA; ^8^ The Canopy Cancer Collective, 1440 Foundation Saratoga California USA

**Keywords:** human‐centered design, learning health network, patient‐centered care, personas

## Abstract

**Introduction:**

Learning health networks (LHNs) improve clinical outcomes by applying core tenets of continuous quality improvements (QI) to reach community‐defined outcomes, data‐sharing, and empowered interdisciplinary teams including patients and caregivers. LHNs provide an ideal environment for the rapid adoption of evidence‐based guidelines and translation of research and best practices at scale. When an LHN is established, it is critical to understand the needs of all stakeholders. To accomplish this, we used ethnographic methods to develop personas of different stakeholders within The Canopy Cancer Collective, the first oncology LHN.

**Methods:**

We partnered with a firm experienced in qualitative research and human‐centered design to conduct interviews with stakeholders of The Canopy Cancer Collective, a newly developed pancreatic cancer LHN. Together with the firm, we developed a personas model approach to represent the wide range of diverse perspectives among the representative stakeholders, which included care team members, patients, and caregivers.

**Results:**

Thirty‐one stakeholders from all facets of pancreatic cancer care were interviewed, including 20 care team members, 8 patients, and 3 caregivers. Interview transcripts were analyzed to construct 10 personas felt to represent the broad spectrum of stakeholders within The Cancer Canopy Collective. These personas were used as a foundation for the design and development of The Cancer Canopy Cancer Collective key drivers and aims.

**Conclusions:**

As LHNs continue to facilitate comprehensive approaches to patient‐centered care, interdisciplinary teams who understand each other's needs can improve Network unity and cohesion. We present the first model utilizing personas for LHNs, demonstrating this framework holds significant promise for further study. If validated, such an approach could be used as a dynamic foundation for understanding individual stakeholder needs in similar LHN ecosystems in the future.

## 1 INTRODUCTION

Navigating the limitations of diagnostic uncertainty, managing the complexity of decision‐making, and adjusting to evolving medical treatments that constantly change over time are challenges that are overcome best by teamwork and collaboration, not by one person alone.^1^ The development of a learning health network (LHN), a collaborative clinical learning community, involves a systematic approach to aligning interdisciplinary teams with science, informatics, and clinical practice resources.^2^, ^3^, ^4^

LHNs hold the potential to improve clinical outcomes through the application of the core tenets of continuous quality improvement (QI), data‐sharing, empowerment of interdisciplinary teams including patients and caregivers, and a focus on community‐defined improvable outcomes.^4^, ^5^, ^6^ Most notably, LHNs provide a person‐centric foundation, as Okun and Goodwin describe, “upon which the knowledge and experience of patients and caregivers are collected, curated, aggregated and shared.”^1^ This in turn supports a dynamic community and organizational structure with input from a team of medical care providers.^5^, ^7^, ^8^

When LHN frameworks are established, it is critical to understand the individual needs of the involved stakeholders. One way to accomplish this is through ethnographic approaches, namely to “provide rich, holistic insights into people's views and actions, as well as the nature of the location they inhabit” through detailed observations and interviews.^9^ In one critical example, Fore et al describe how “goal‐directed design” methods, including ethnographic and observational approaches, were successfully employed to understand the unique circumstances of potential participants in a learning health system model designed specifically for pediatric inflammatory bowel disease.^10^ Their approach allowed for the design of a network of healthcare interventions that reflect the purpose of their collaborative care network in pediatric chronic illness care.^10^

To date, these methods have not been translated to LHNs that exist in the oncology space, which bears a unique set of real‐life challenges and perspectives. In this report, we document the application of human‐centered design principles used in the development of The Canopy Cancer Collective,^11^ a newly developed LHN for pancreatic cancer. To assess perspectives within the LHN, stakeholders within The Canopy Cancer Collective were grouped thematically into “personas.” Personas are fictional, composite representations of a sample of real people that bring a humanistic image and identity to stakeholder or “user” types. They serve to create a memorable, engaging, and actionable image that serves as a target of design efforts.^1^, ^12^ Personas convey information about stakeholders in ways other modalities cannot, allowing for the overall design to become more human‐focused.^12^

Once created, the personas were shared among the LHN leadership to launch a QI process targeted at leveraging personas in this particular LHN design. The aim of this paper is to demonstrate how the utilization of personas aided in the development of our interdisciplinary LHN key drivers and to provide qualitative insights from this human‐centered design approach.

## 2 QUESTIONS OF INTEREST

How do we ensure that a LHN is designed to engage with all its stakeholders? In this study, we propose using a personas model approach to represent a wide range of diverse perspectives within a Network ecosystem.

## 3 MATERIALS AND METHODS

### 3.1 The canopy cancer collective

The Canopy Cancer Collective consists of a collaboration among 14 cancer institutes across the United States aimed at improving healthcare delivery to patients with pancreatic cancer. Each participating healthcare system employs accomplished medical care providers, cancer researchers, and wellness specialists to formulate and foster best practices in care management to accelerate improvements in outcomes in pancreatic cancer.

### 3.2 Persona development

**FIGURE 1:**
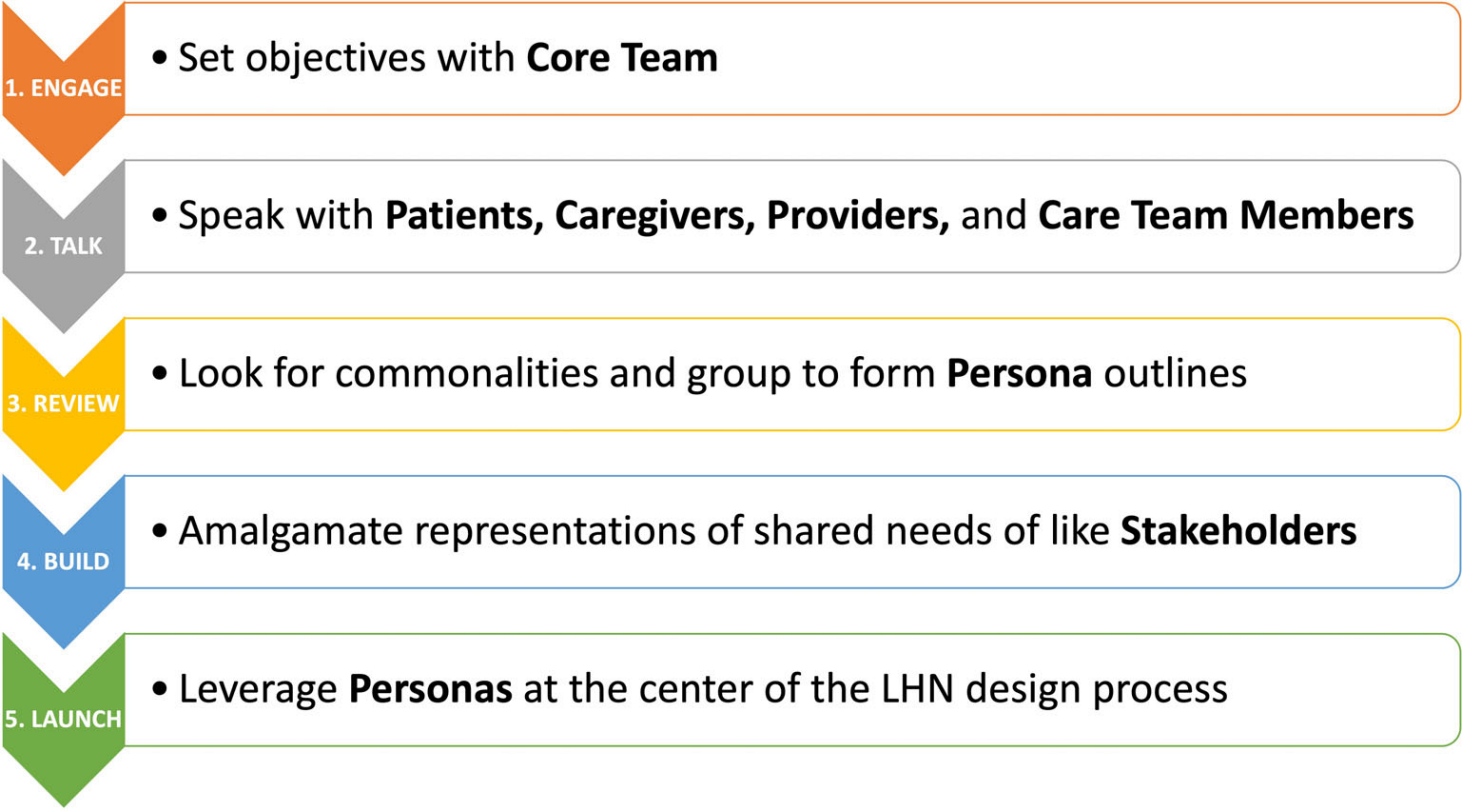
5‐Step process of development and application of personas.

Personas were used to guide the development of The Canopy Cancer Collective by providing relevant, actionable insights throughout its formation. Development of personas was executed in a 5‐step process, as shown in Figure 1.

### 3.3 Step 1: setting objectives

Once target interview groups were created in March 2020, interviewees from the initial cohort of 6 academic sites affiliated with The Canopy Cancer Collective were recruited and dates for remote interviews were scheduled. A core team, composed of 6 physician champions, one from each site, was engaged to set objectives for how the personas would be developed and used.

### 3.4 Step 2: stakeholder interviews

Written by the physician champions with support from the SEEK Company (Cincinnati, Ohio), a qualitative insights and innovation consultancy firm, an interview guide (Figure S1) was developed to focus the patient, caregiver, and care team member interviews.

Patients and caregivers were interviewed in their home environment, which added context to spoken needs and highlighted motivations and barriers to improvement that are otherwise invisible to or not fully appreciated by the clinician. Additionally, private interviews with care team members were conducted in the clinical setting, and inputs from all participant groups were extracted from the interview transcripts.

Two members of the consultancy firm spoke to patients and caregivers from a wide variety of backgrounds and treatment stages to understand their journeys, needs, hurdles, motivations, and goals. They also engaged providers from all aspects of the treatment process to understand their perspectives, treatment approaches, needs, hurdles, motivations, and goals.

### 3.5 Step 3: qualitative data collection

Subsequently each of the 6 physician champions, along with support from SEEK Company consultants, reviewed interview transcripts and notes to look for commonalities in needs, motivations, goals, and hurdles among stakeholders, and grouped them to form persona outlines. Interview transcripts were analyzed to find thematic patterns among stakeholders. Thematic categories were developed according to stakeholder responses and inputted into a spreadsheet shared among the physician champions and consultants. This step was then repeated for caregivers and care team members as well.

### 3.6 Step 4: construction of personas

Following several rounds of discussion and feedback, personas were constructed as thematic representations of shared needs, wants, and points of tension among involved stakeholders. The core team of physician champions determined that 10 personas would be the ideal number to best capture the gamut of stakeholders involved in the LHN. Once the personas were formed and visually illustrated, they were shared with stakeholders who had expressed interest in offering iterative feedback on the rollout of the personas to counter potential biases and ensure their validity. This subset of stakeholders participated in group sessions with the physician champions to allow for open feedback regarding the personas' ability to best represent stakeholder needs, motivations, and overall goals.

### 3.7 Step 5: leveraging personas for the LHN

Stakeholder comments and feedback were shared with the LHN core team to finalize the personas. Once completed, design meetings were held to discuss how well the personas matched the representative needs of each stakeholder at individual LHN sites and how geographic settings may impact the different personas at each participating site within The Canopy Cancer Collective. An action plan was generated to use the personas to develop the key aims and drivers of The Canopy Cancer Collective.

### 3.8 Ethics statement

This study reports on data collected from humans. Institutional Review Board approval was not required because this analysis is exempt from the regulatory review requirements as set forth in section 46.101 (b) of 45 CFR 46: Research involving the collection or study of existing data, documents, records, pathological specimens, or diagnostic specimens, if these sources are publicly available or if the information is recorded by the investigator in such a manner that subjects cannot be identified, directly or through identifiers linked to the subjects.

## 4 RESULTS

### 4.1 Stakeholder characteristics

In total, 31 stakeholders were interviewed from across the initial six academic sites affiliated with The Canopy Cancer Collective. Interviewees included 20 care team members, eight patients, and three caregivers. Of the 20 care team members, there were two medical oncologists, two surgical oncologists, two radiation oncologists, two physician assistants, three nurses, a dietitian, a therapeutic gastroenterologist, and a social worker. The remaining six care team members were administrators, including two scheduling coordinators, two administrative assistants, a director of nurse navigation, and a director of LHN operations. The eight patients included men and women of various ages and demographic backgrounds with a history of pancreatic cancer. Of the caregivers interviewed, one was the adult child of a patient while the remaining two were patient partners.

### 4.2 Presenting our personas

Ten composite personas representing The Canopy Cancer Collective emerged from the interviews (Figure S2). Brief descriptions of each persona and its corresponding sets of roots—beliefs, foundations, and behaviors that are deeply embedded into one's role within the LHN—and needs are shown in Table 1.

**TABLE 1:** Personas with corresponding roots and needs.

Stakeholder	Persona	Roots	Needs
Patient	*Stunned and Struggling*	Core instability, survival, control	Connection, empowerment, support
	*At the Helm*	Control, communication, trust	Collaboration, transparency, educational resources
	*It Takes a Village*	Connection, generation, self‐realization	Trusting environment, mutual understanding
Caregiver	*The Gentle Giant*	Connection, expression, realization	Acknowledgment, recognition, support
MDC director (Radiation Oncologist)	*Advocate Gatherer*	Unity, momentum, traction	Efficiency, clinical “wins,” cross‐pollination opportunities
MDC nurse	*Between Me and Care*	Actualization, generation, communication	Reduce burdens, clear roles, standard communication tools
Medical oncologist	*The Relentless Fighter*	Mastery, unity, balance	Open‐handed collaboration, creative solutions, faster path to MDC
Surgical oncologist	*Knife's Edge*	Stability, connection, expression	Faster path to MDC, open debate, dedicated patient support
Social worker	*Working From the Outside In*	Connection, control, expression	Inclusion, access and time with patients, support from the MDC
Scheduling coordinator	*Fitting It All In*	Coordination, control, communication	Efficiency, codification of workflow, access to outside records

These personas highlight the wide variation among patient, caregiver, and care team perspectives. One example of this is highlighted by a comparison of “Stunned and Struggling” with “At the Helm.” While the former patient is overwhelmed by the range of new information, stresses, and emotions involved with a cancer diagnosis to the point of near paralysis, the latter patient's reaction is to focus on what can be controlled and the fight to come. These two patients respond differently to interactions within the clinic and their home environment, and correspondingly the interaction between staff and each of these patients must be tailored appropriately.

### 4.3 Impact of personas

Once the personas were constructed, The Canopy Cancer Collective leadership team reviewed the most represented needs. Communication, collaboration, and control were each a component of 40% of the personas, and 30% of the personas incorporated expression and self‐realization/actualization.

In sharing and discussing these personas, themes of open‐mindedness and mutual support were brought to the forefront among core members of the LHN. Acknowledgment and recognition of the different perspectives within the LHN became critical inputs to designing Network aims and goals.

On the patient and caregiver side, we found a one‐size‐fits‐all approach cannot be applied. While some patients and caregivers may prefer to take full ownership of their care, others may retreat from their diagnoses. This dichotomy is crucial for providers to understand before initiating treatment planning conversations and may be assessed by shared decision‐making tools and surveys. Without paying close attention to how each patient may fit into potential personas, the provider can lose patient trust, the foundation of the patient–provider relationship.

Additionally, while the caregiver persona has not been well‐characterized, the persona (“The Gentle Giant”) was insightful in recognizing that the experience of a pancreatic diagnosis can be just as—if not more—triggering for a caregiver than the patient. Helping create a safe space for caregivers and patients alike to ask the questions that are important to them will make them feel empowered to speak up to optimize their outcomes.

In the setting of tumor boards and interdisciplinary discussions, the care team personas that are either more senior or more outspoken have the potential to drive the treatment decisions or pathways. In some cases, this is reasonable, but if the team does not proactively elicit feedback from more introverted personas, it may miss out on important feedback that is integral for the success of patient outcomes and treatment plans. Creating a safe and open environment where all voices can be heard is crucial.

When building an LHN, understanding stakeholder perspectives and experiences is an important first step in creating a collaborative environment so every individual across the interdisciplinary team can provide individual input to improve the Network, which can have an exponential effect on patient care. This allows everyone, regardless of position or rank, to feel comfortable sharing their perspectives.

**FIGURE 2:**
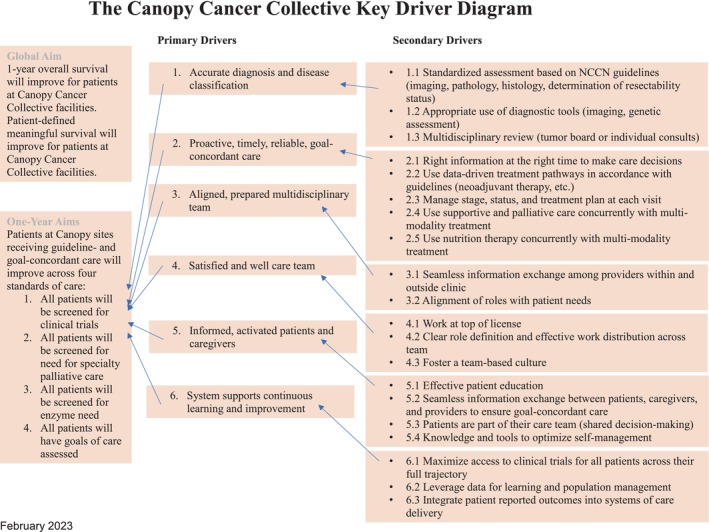
The Canopy Cancer Collective key driver diagram.

Lastly, the key drivers of The Canopy Cancer Collective were guided by discussions around our personas model (Figure 2). For example, these discussions emphasized the importance of input from the patient and caregiver (Primary Driver 5) and the importance of shared decision‐making. Another product of these discussions was a renewed focus on healing and quality of life of the providers (Primary Driver 4) as well as patients through the promotion of open‐ended communication practices, mindfulness exercises, and support groups.

## 5 DISCUSSION

LHNs provide an ideal environment for the rapid adoption of evidence‐based guidelines and translation of research and best practices at scale.^13^ As LHNs continue to facilitate holistic approaches to patient‐centered care, interdisciplinary clinic members should try to understand each other's needs so that the team can be unified in patient care.^14^ Personas have been used and applied in several industries, such as design, gaming, software development, marketing, and media, but this is one of the first applications, to the best of our knowledge, of personas in the development of a cancer LHN ecosystem.^10^, ^15^

By leveraging the development of personas, we can chart strategies for how to engage the network in a shared understanding of the unique experiences and challenges of each care team member, patient, and caregiver, so Network members can tailor healthcare interactions accordingly. Following further testing, this model can potentially lead to improvements in the efficiency of healthcare delivery and a more well‐rounded LHN.^16^, ^17^ One future direction is the creation of a patient clinical profile that contains the individual's health history, which can be compared to close clinical matches archived in an LHN database. The care team may then sort and prioritize treatment regimens based on clinical matches and present a consensus review with holistic treatment recommendations tailored to the individual patient.

Recently, Ennis and Vapiwala described cognitive biases that may impact clinical practice, and research into methods to overcome these biases is ongoing.^18^ This project brought to light how critical it is to recognize our unconscious biases and consider how different personas may tie into those biases. This is impactful for realizing the full potential of all input across interdisciplinary teams and minimizing disparate treatment decisions due to implicit provider bias. This was especially salient as our LHN began development during the initiation of the COVID‐19 pandemic with virtual communication between stakeholders. Grounding our LHN in human‐centered design was even more important in fostering strong connections within the LHN during its developmental stages.

By exploring the diversity of LHN stakeholder perspectives, the personas model can create a common language with which to meaningfully discuss patient care matters. It can engender both interest and empathy toward involved stakeholders, engaging the LHN team in a way that other representations cannot. This allows for LHN buy‐in on how to best support one another, creating a shared understanding of stakeholder goals.

Previous studies report various benefits associated with persona application, including alignment of stakeholder understanding and communication within a team, increasing empathy and user‐centricity, and avoiding self‐centered bias in product design and development activities.^14^, ^19^ However, as far as studies of similar development for cancer LHNs are concerned, the possibilities for comparison are limited. While there are numerous studies addressing the development of LHNs generally, these focus more on theory and policy and other application areas but do not discuss ethnographic approaches to LHN design.^5^, ^20^, ^21^, ^22^, ^23^, ^24^, ^25^ As the first personas model reported for cancer LHNs, we plan to prospectively validate the use of this model for more effective interdisciplinary care collaboration. It is our hope our personas can be adapted and used as a dynamic foundation for other interdisciplinary LHN frameworks.

We recognize there are numerous limitations to the application of personas. Critics argue personas lack methodological robustness, involve small sample sizes, and have unproven use cases and benefits.^14^, ^26^, ^27^, ^28^ Additionally, our design is limited by its inability to truly cover the diversity of patients, caregivers, and care team members of our LHN. Creating a clinical environment or healthcare structure—for example, a single‐day multidisciplinary clinic or multiple visits at different sites—that will serve every different persona is impossible.^29^ While we cannot create an operational structure to support every type of patient, we can personalize information given to patients and caregivers based on the wishes that are most aligned with their persona at the point of care. Additionally, the core team that finalized the personas was composed only of physician champions. Though other stakeholder groups offered feedback on the personas that were developed, the absence of these groups may have led to biases in the final rollout of these personas. Lastly, one of the risks of personas is that stakeholders are generally more nuanced than their persona representations, which do not account for the full complexity and dynamism of stakeholder needs over time. Personas should therefore be viewed as a valuable learning and training exercise for LHN development that warrants iterative testing and further validation.

## 6 CONCLUSIONS

LHN frameworks and approaches remain critical to ensuring continuous innovation within organizations and collaborative data application based on a shared vision. To make the most of the LHN, it is critical to elicit the perspectives and needs of involved stakeholders. We queried patients, caregivers, and care team members about their experiences and clinical perceptions to generate personas, which identified several areas for continued investigation. These areas include a need for improved connection between stakeholders, open‐ended collaboration among care team members, and improved communication strategies.

Our work demonstrates how the collection of qualitative data can be leveraged to create biopsychosocial personas to explore and address the diversity in behaviors, preferences, and needs in interdisciplinary teams. The development of personas for LHNs has the potential to ensure that an LHN is designed to engage with all of its stakeholders and improve the lives of patients. Further research into validating this approach and developing implementation strategies is warranted. Our results lay the foundation for the translation of qualitative approaches to LHNs that service pancreatic and other complex malignancies that will benefit from transformative, system‐based approaches to improve outcomes.

## CONFLICT OF INTEREST STATEMENT

Joseph Herman reports consulting for Boston Scientific and consulting and stock ownership at Histosonics. All affiliate sites receive research funding from The Canopy Cancer Collective and 1440 Foundation. There are no other conflicts to disclose related to the material discussed in this manuscript.

## Supporting information


**Figure S1.** Interview guide for patients and care team members.

**Figure S2.** Presentation of The Canopy Cancer Collective personas.
