# Supplementary material for: The power of personas: Exploring an innovative model for understanding stakeholder perspectives in an oncology learning health network
Source: Learn Health Syst. 2024 May 27;9(1):e10422. doi: 10.1002/lrh2.10422 (PMC11733431; doi:10.1002/lrh2.10422)
Supplement: Supplementary file 1 — Figure S1. Interview guide for patients and care team members. [file LRH2-9-e10422-s001.docx]

**Supplemental Figure 1:** Interview Guide for Patients and Care Team Members

| **Patient Interviews** |
| --- |
| **Life Before Pancreatic Cancer**  · Demographic information  · What are your current goals? Motivations? Successes?  · Do you have any additional support in completing daily tasks or chores? |
| **Medical History and Perception of Condition**  · Could you share more about your health history?  · What were your first symptoms that led you to pursue medical involvement?  · How were you first diagnosed with pancreatic cancer?  · What was it like seeing different physicians and getting different perspectives?  · What did you know about pancreatic cancer before you were diagnosed?  · What were the treatment options presented to you?  · What was it like learning about the staging process?  · Could you share any hiccups in your cancer journey? Any experience with treatment progression? |
| **Relationship to Care Team**  · What was the transition like from seeing your PCP to Cancer Care?  · Did you see surgical, medical, and radiation oncology?  · How often did you see each oncologist?  · What was your relationship like with the nursing staff?  · What was your experience with the support staff?  · Did you face any difficulties accessing health services? |
| **Current Life with Pancreatic Cancer**  · Can you tell me more about your daily journey with Pancreatic Cancer?  · Can you share what the impact of your treatment has been like?  · What are coping behaviors you may have developed during your treatment?  · Can you identify some barriers to daily success?  · Can you share victories/“wins”/moments of encouragement throughout your care?  · What about some losses/setbacks/moments of disappointment?  · Can you share frustrations with care today? Any opportunities for improvement? |
| **Looking Forward**  · What are your future goals and motivations moving forward?  · What are your hopes for the future of Pancreatic Cancer care for future patients? |

| **Care Team Interviews** |
| --- |
| **Pancreatic Cancer Landscape**  · Demographic information  · What is the nature of pancreatic cancer vs. other cancers?  · What are the options in pancreatic cancer care?  · What are some new pancreatic cancer approaches? |
| **Motivations and Drivers**  · What is your philosophical approach to current treatment?  · What are your goals specifically?  · What are balancing and mitigating factors in Pancreatic Cancer care?  · What are the hallmarks of success?  · What about the hallmarks of failure? |
| **Opportunities and Outages**  · What are current opportunities in pancreatic cancer care that exist today that may not have existed 10 years ago?  · What are frustrations and missing elements in Pancreatic Cancer care?  · What are encouraging developments in Pancreatic Cancer care? |
| **Looking Forward**  · What are your hopes for the future of Pancreatic Cancer care?  · What are your future professional goals and motivations moving forward?  · What are your frustrations with care today?  · What are opportunities for improvement? |
