# Supplementary figures and images for: The power of personas: Exploring an innovative model for understanding stakeholder perspectives in an oncology learning health network
Source: Learn Health Syst. 2024 May 27;9(1):e10422. doi: 10.1002/lrh2.10422 (PMC11733431; doi:10.1002/lrh2.10422)

**Supplemental Figure 2:** Presentation of The Canopy Cancer Collective Personas **
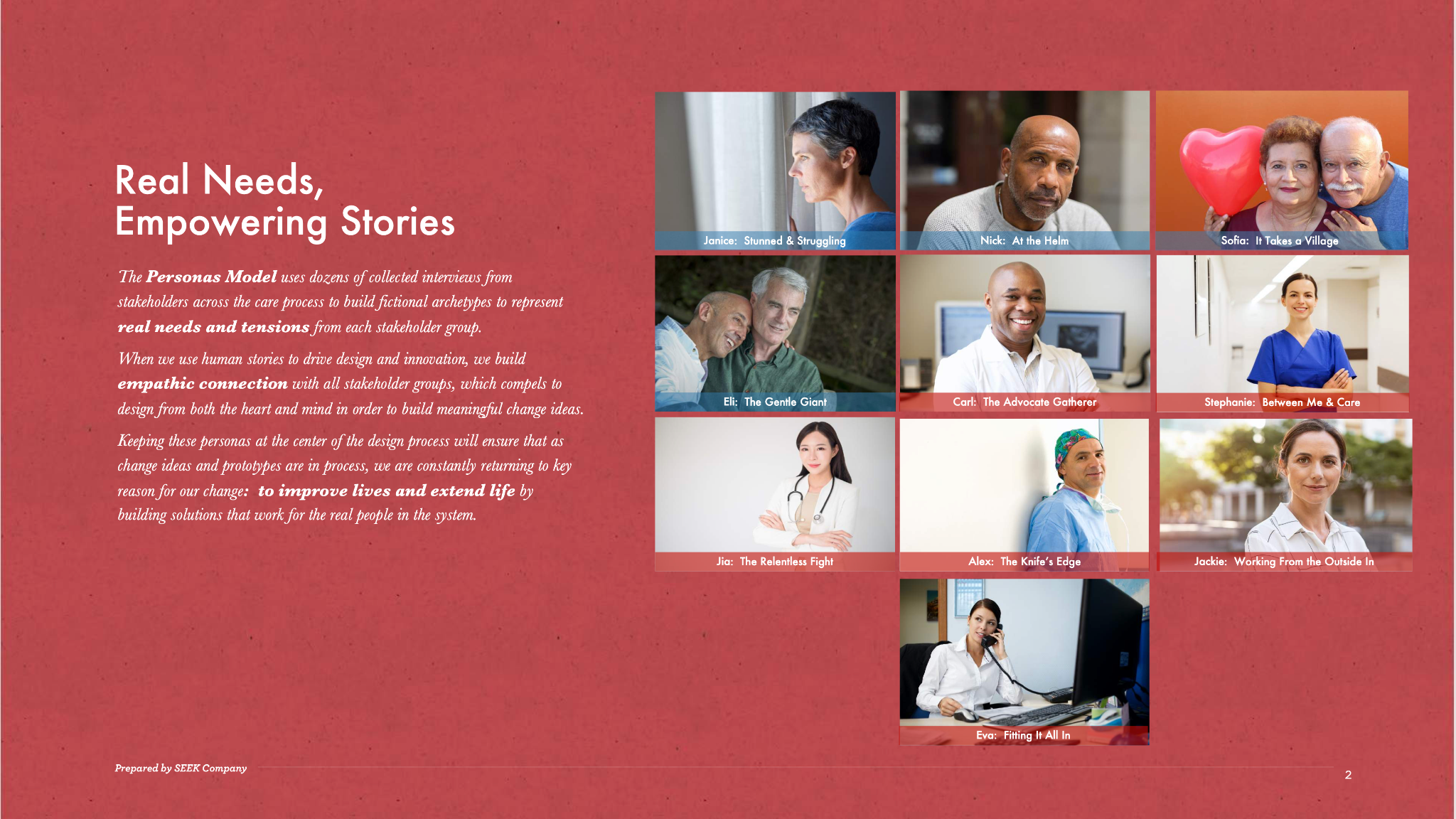
**


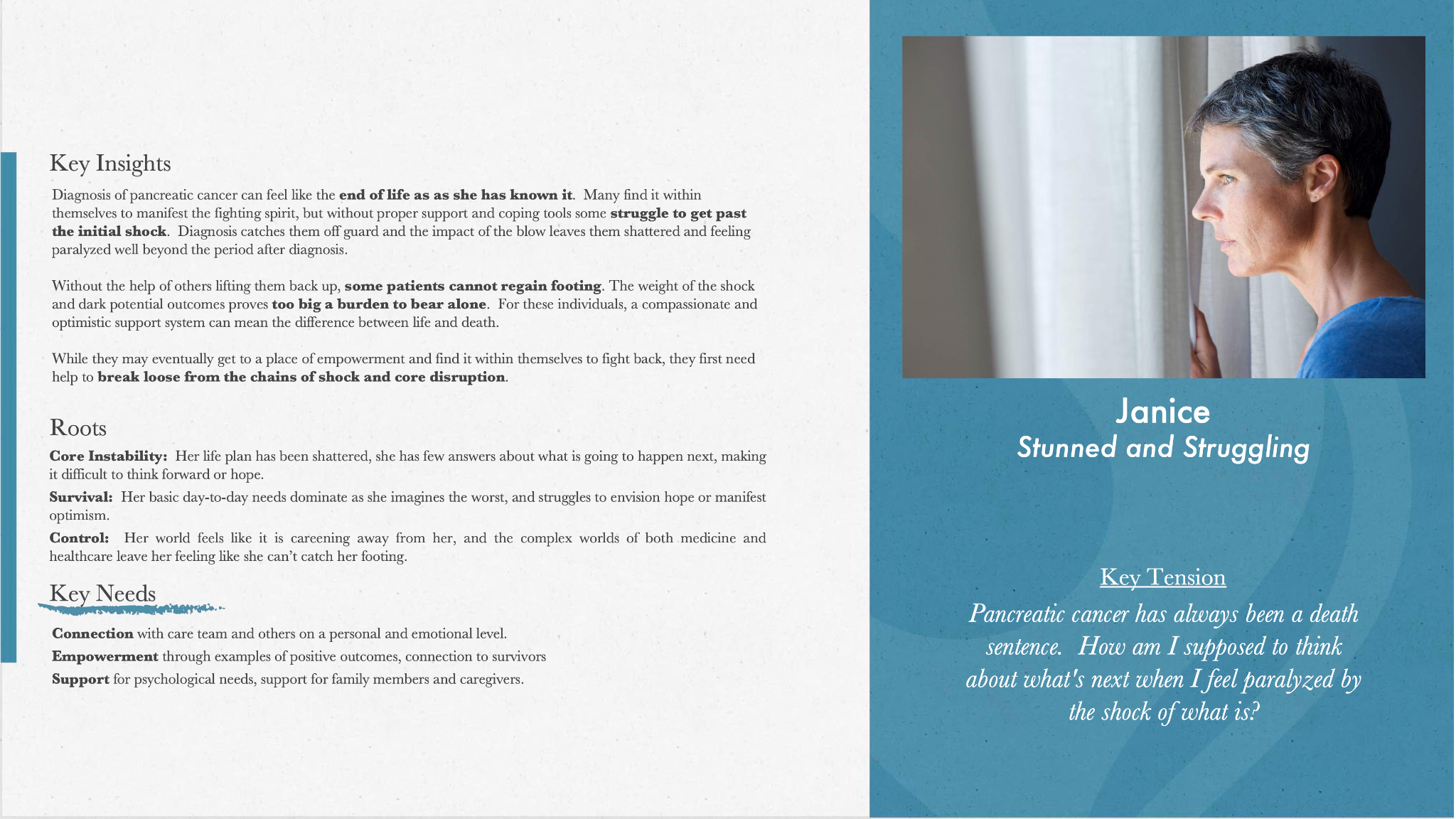

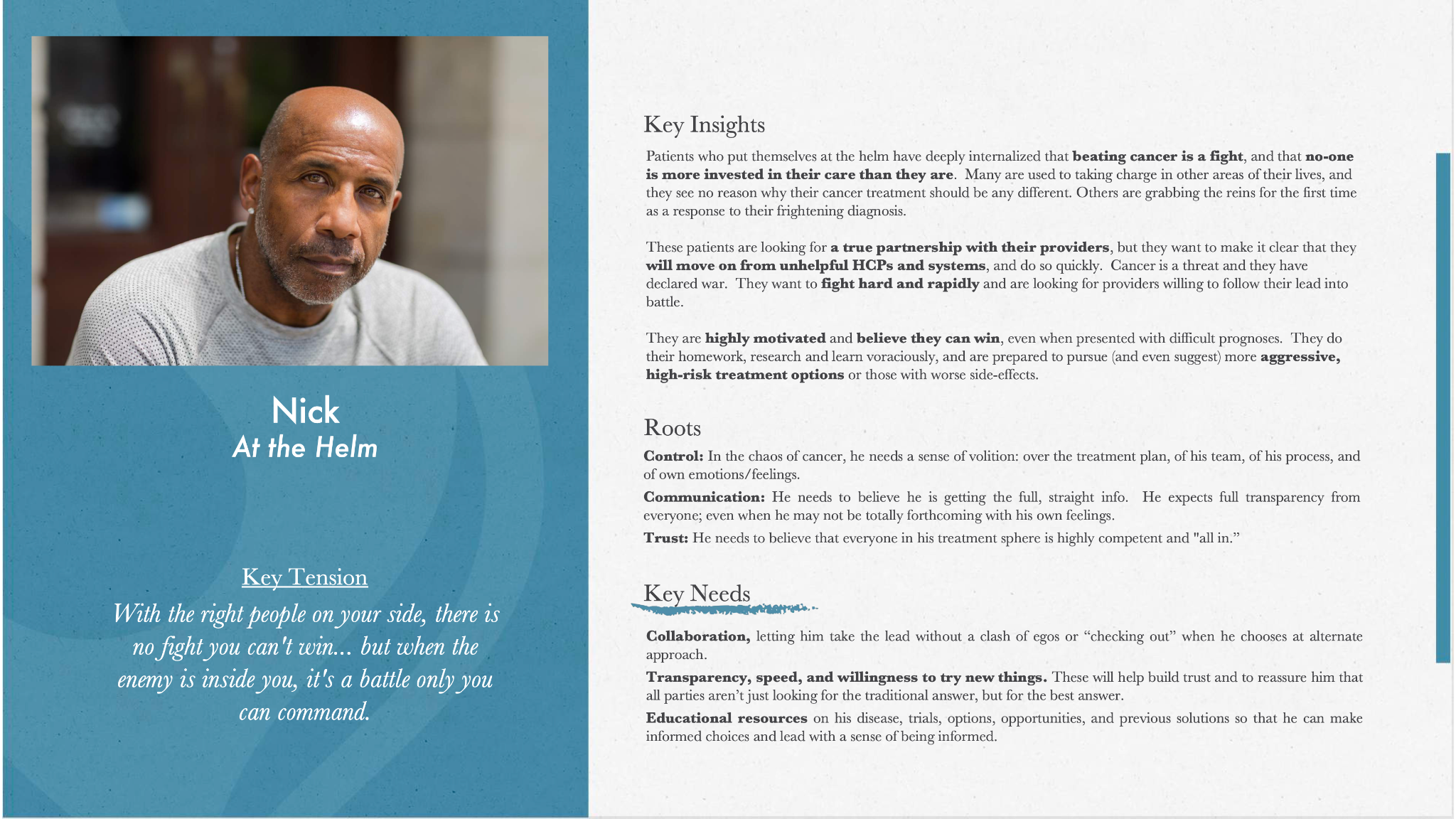


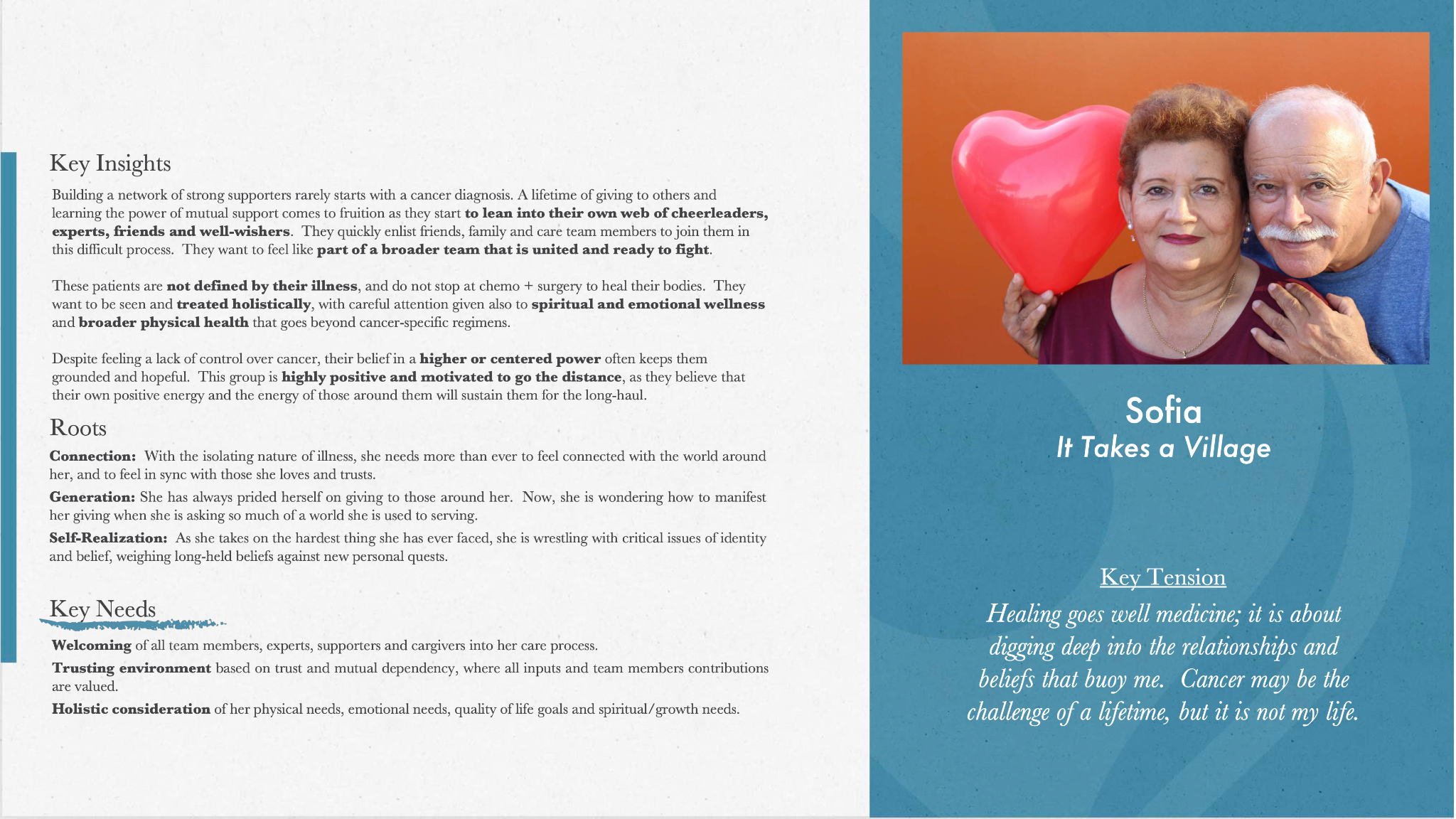


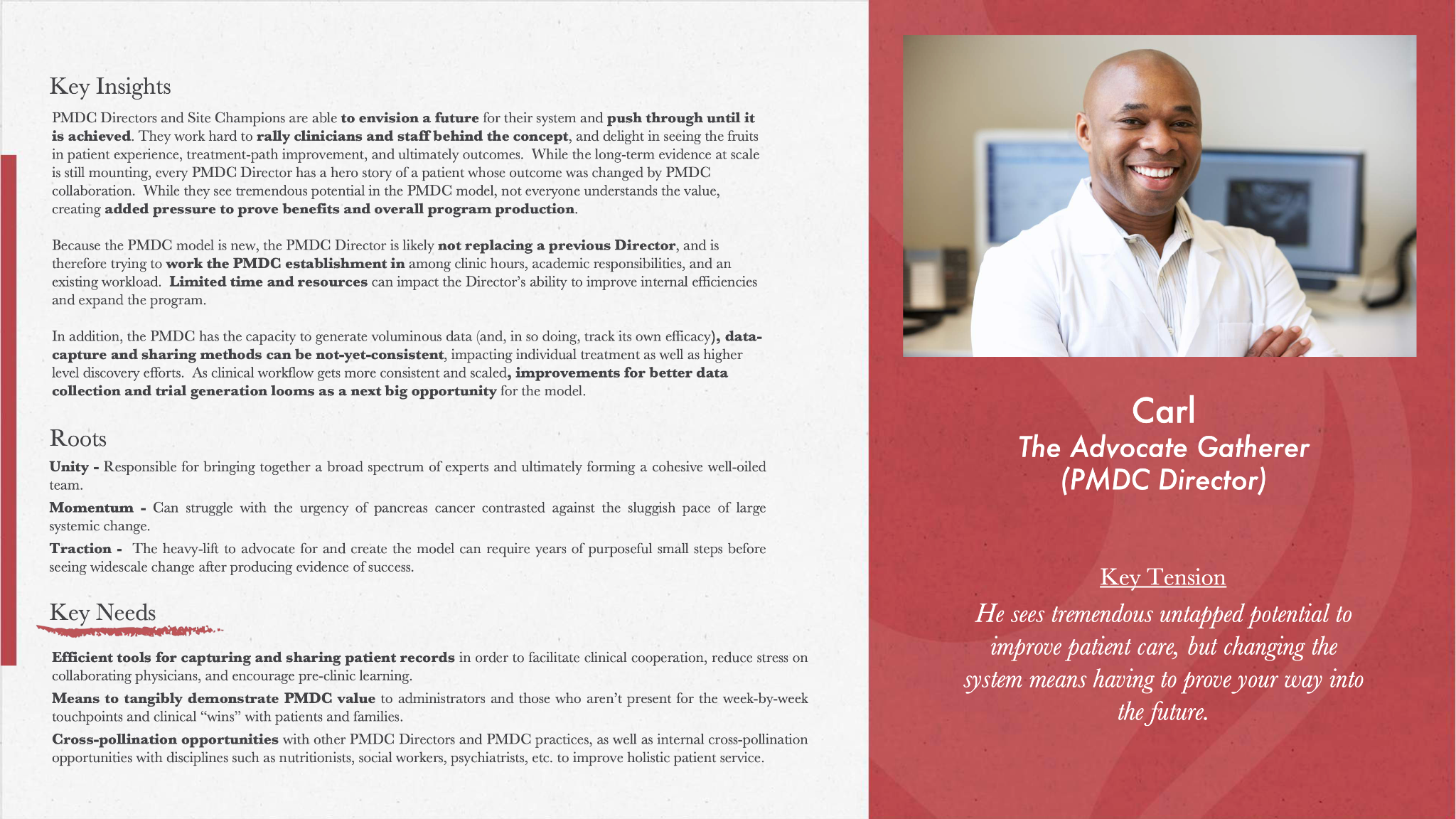


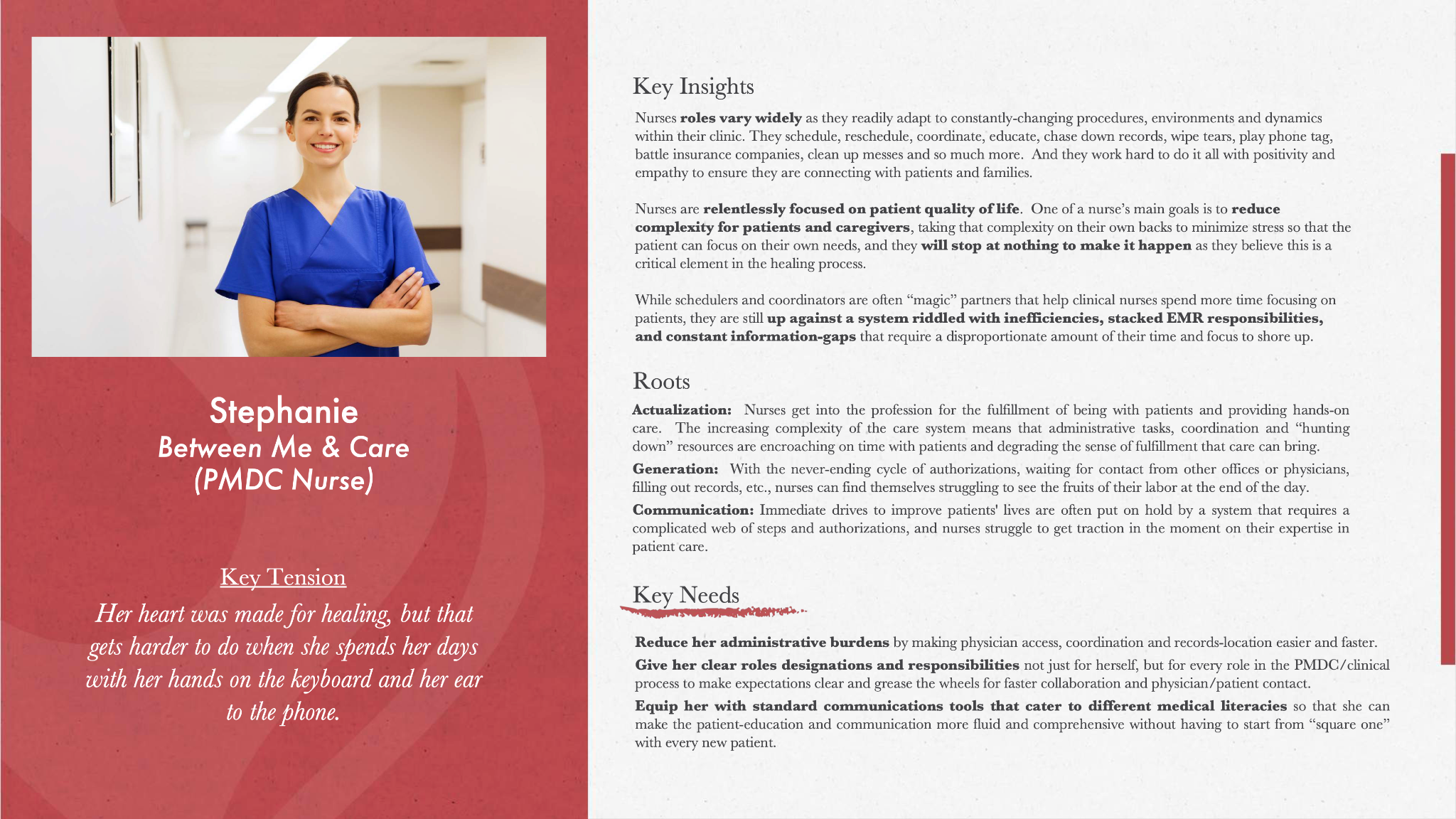


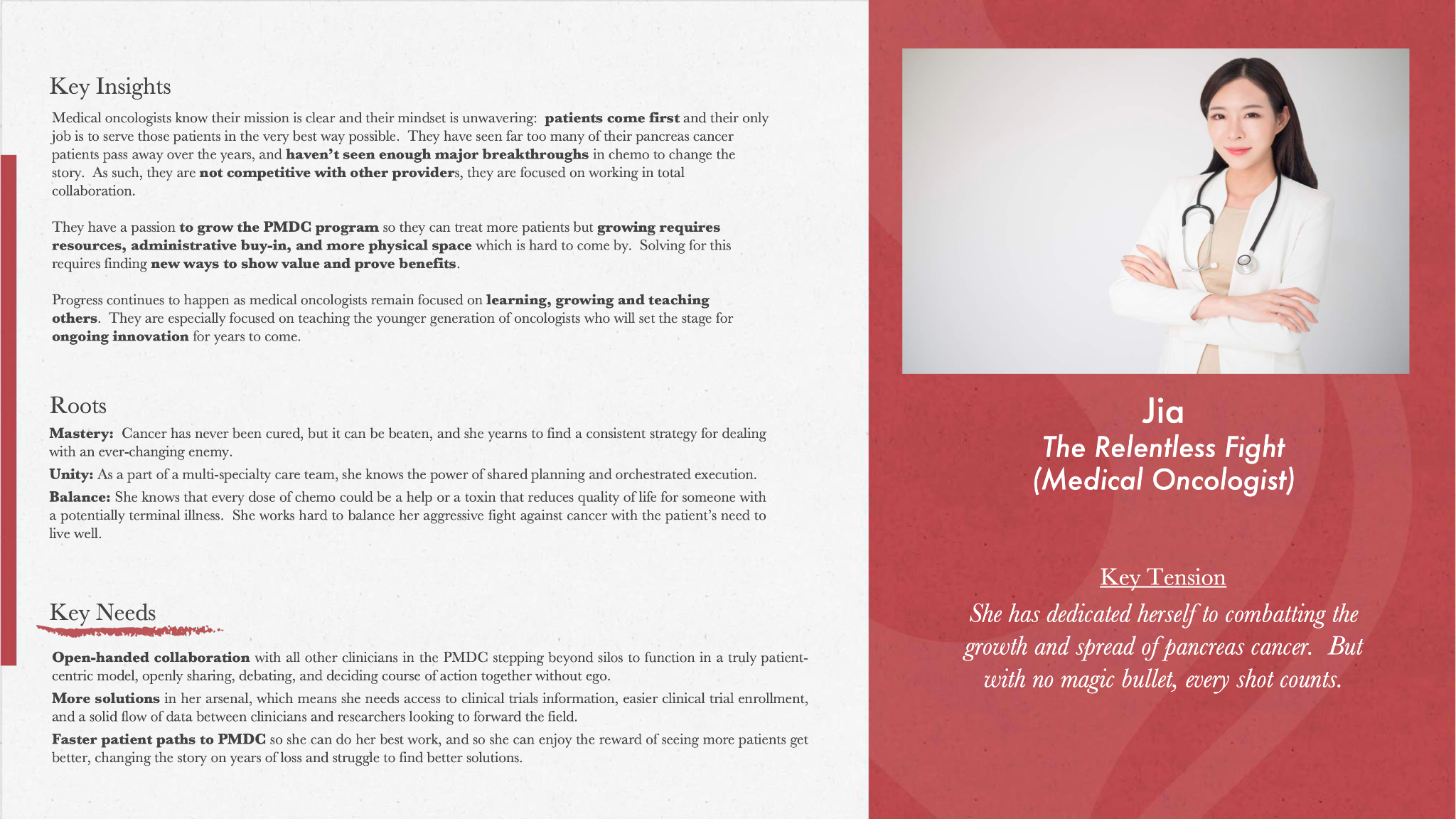


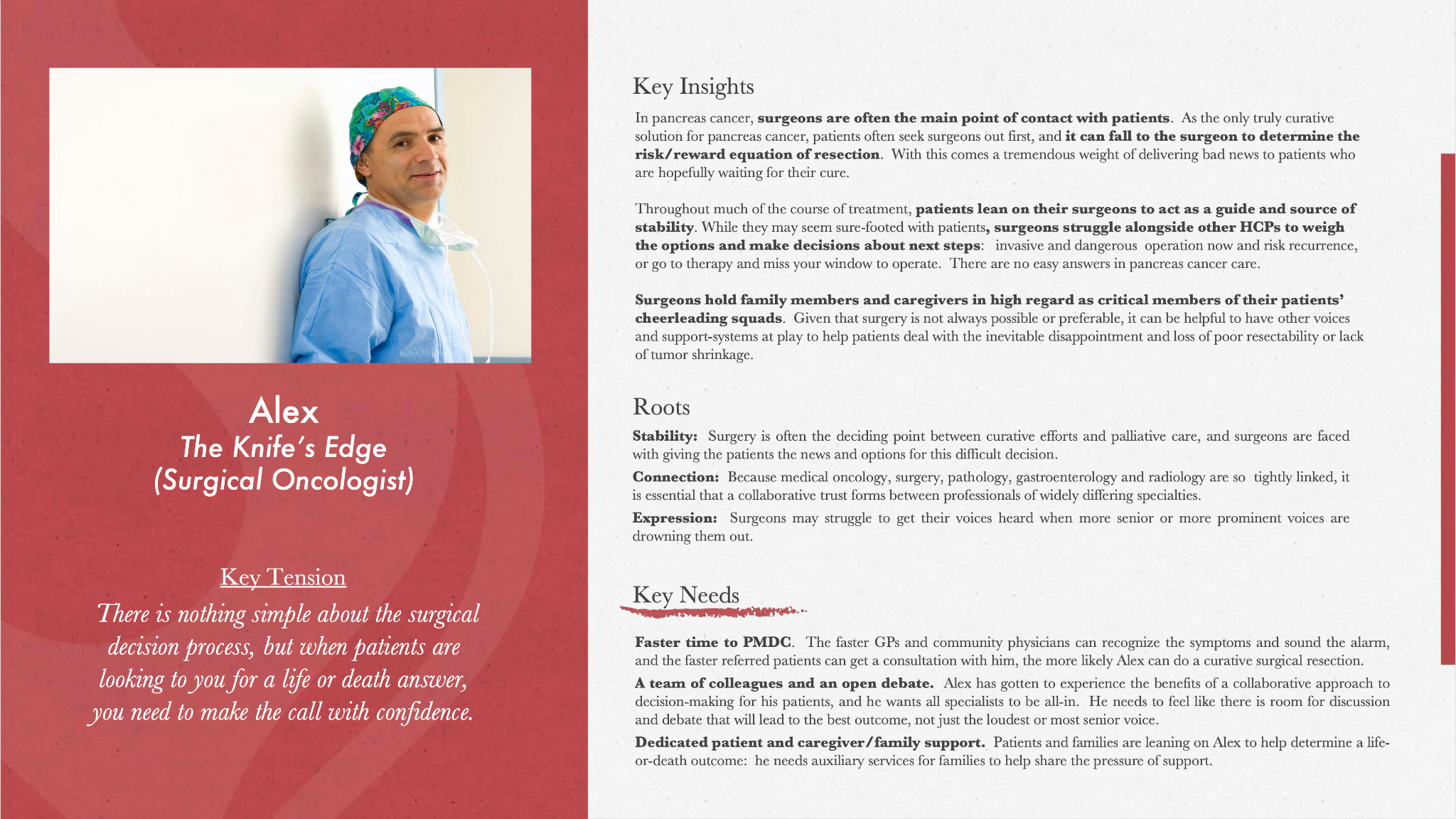


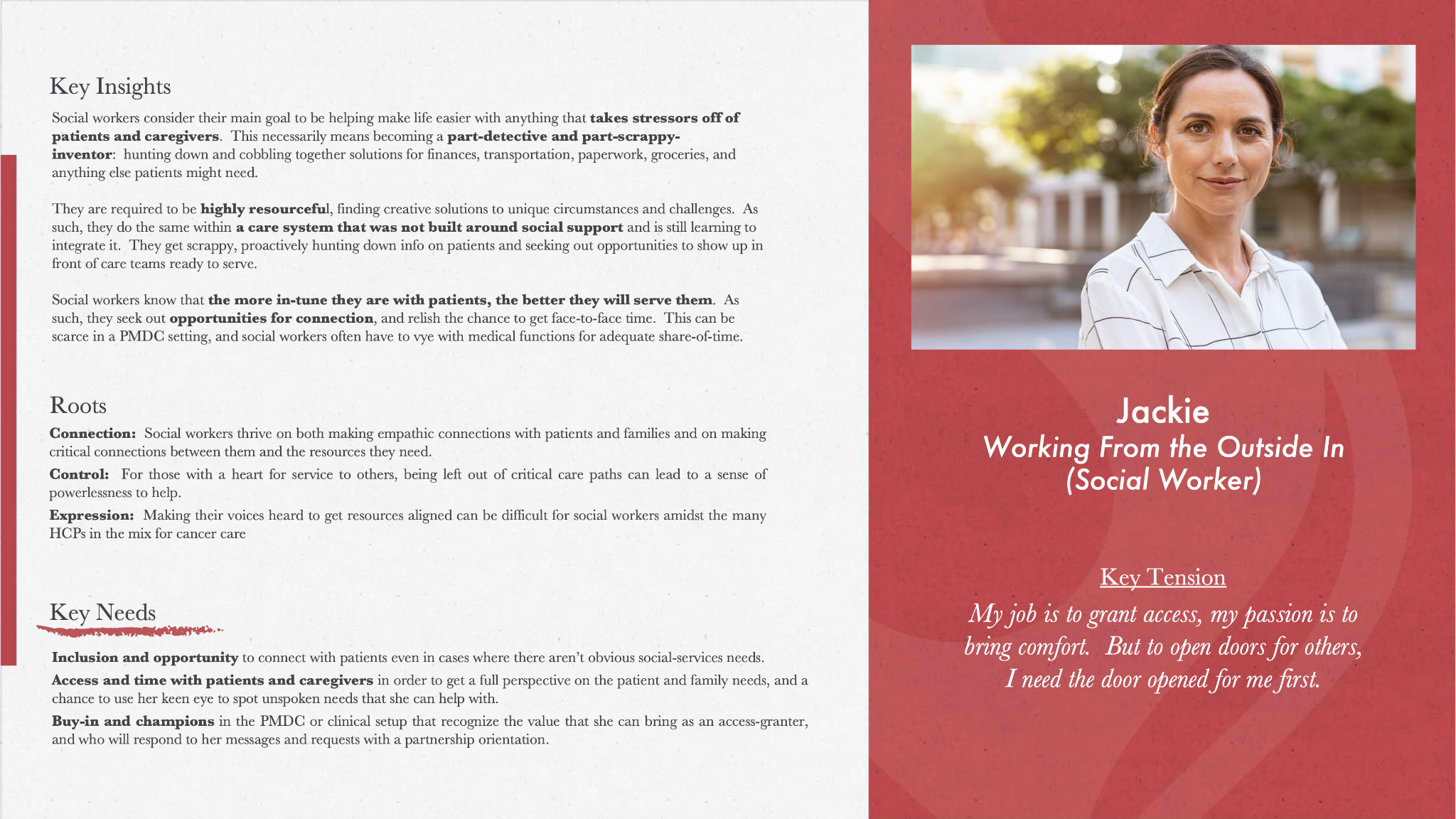


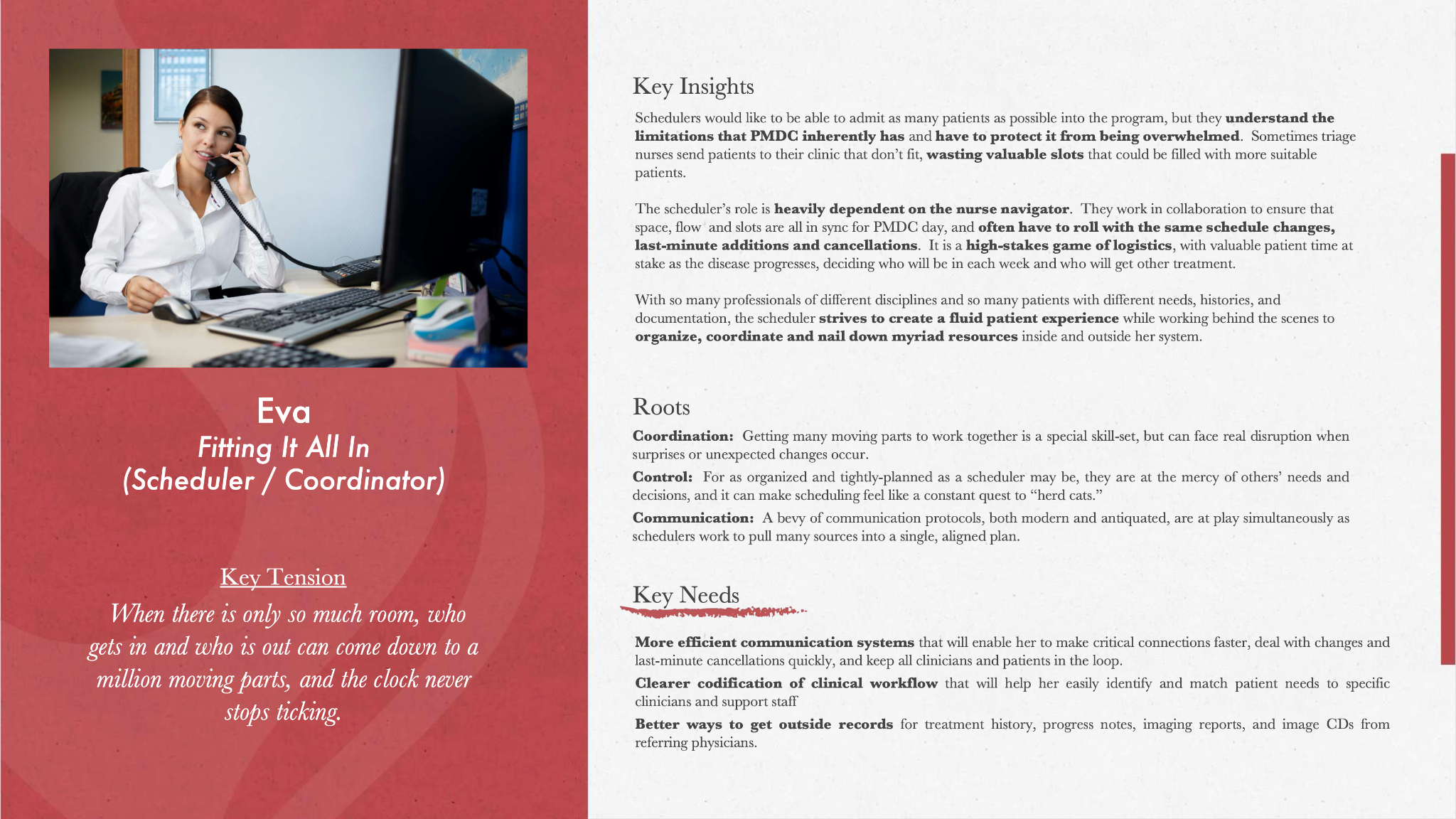

Supplement: Supplementary file 2 — Figure S2. Presentation of The Canopy Cancer Collective personas. [file LRH2-9-e10422-s002.docx]
